# Blood Eosinophils and Clinical Outcomes in Patients With Acute Exacerbation of Chronic Obstructive Pulmonary Disease: A Propensity Score Matching Analysis of Real-World Data in China

**DOI:** 10.3389/fmed.2021.653777

**Published:** 2021-06-09

**Authors:** Yanan Cui, Zijie Zhan, Zihang Zeng, Ke Huang, Chen Liang, Xihua Mao, Yaowen Zhang, Xiaoxia Ren, Ting Yang, Yan Chen

**Affiliations:** ^1^Department of Respiratory and Critical Care Medicine, The Second Xiangya Hospital, Central South University, Changsha, China; ^2^Department of Pulmonary and Critical Care Medicine, China-Japan Friendship Hospital, Beijing, China; ^3^National Clinical Research Center for Respiratory Diseases, Beijing, China; ^4^Institute of Respiratory Medicine, Chinese Academy of Medical Science, Beijing, China; ^5^Chinese Alliance for Respiratory Diseases in Primary Care, Beijing, China

**Keywords:** AECOPD, eosinophils, smoking, prediction, corticosteroids

## Abstract

**Background and Objective:** Elevated eosinophils in chronic obstructive pulmonary disease (COPD) are recognized as a biomarker to guide inhaled corticosteroids use, but the value of blood eosinophils in hospitalized exacerbations of COPD remains controversial. This study aimed to evaluate the accuracy of eosinophils in predicting clinical outcomes in acute exacerbation of COPD (AECOPD).

**Methods:** We analyzed data from the acute exacerbation of chronic obstructive pulmonary disease inpatient registry (ACURE) study, which is an ongoing nationwide multicenter, observational real-world study in patients admitted for AECOPD. Data collected between January 2018 and December 2019 in 163 centers were first reviewed. The eligible patients were divided into eosinophilic and non-eosinophilic groups, according to blood eosinophil with 2% of the total leukocyte count as the threshold. Propensity score (PS) matching was performed to adjust for confounders.

**Results:** A total of 1,566 patients (median age: 69 years; 80.3% male) were included and 42.7% had an eosinophilic AECOPD. Eosinophil count <2% was associated with the development of respiratory failure and pneumonia. After PS matching, 650 pairs in overall patients, 468 pairs in patients with smoking history and 177 pairs in patients without smoking were selected, respectively. Only in patients with smoking history, the non-eosinophilic AECOPD was associated with longer median hospital stays (9 vs. 8 days, *P* = 0.034), higher dosage of corticosteroid use, higher economic burden of hospitalization, and poorer response to corticosteroid therapy compared to the eosinophilic AECOPD. No significant difference was found in patients without smoking. Eosinophil levels had no relationship with the change of COPD Assessment Test scores and readmissions or death after 30 days.

**Conclusion:** Elevated eosinophils were associated with better short-term outcomes only in patients with a smoking history. Eosinophil levels cannot be confidently used as a predictor alone for estimating prognosis.

## Introduction

Chronic obstructive pulmonary disease (COPD) is a major cause of morbidity and mortality throughout the world that incurs a considerable economic and social burden (1). Acute exacerbation of COPD (AECOPD) is an acute event characterized by a worsening of respiratory symptoms and leads to a change in therapy (2). AECOPD requiring hospitalizations are associated with an accelerated decline in lung function, a higher probability of recurrent exacerbations, an increase in healthcare costs and mortality (3). The identification of phenotypes based on biomarkers could help clinicians to establish individual treatment programs for patients with AECOPD.

Blood eosinophils are recommended by the Global Initiative for Chronic Obstructive Lung Disease (GOLD) 2020 as a biomarker to guide clinical decisions about inhaled corticosteroids (ICS) use (1). Sources of evidence include *post-hoc* analyses and pre-specified analyses, which showed a better response to ICS in stable COPD patients with higher blood eosinophil counts. However, cohort studies investigating the relationship between blood eosinophils and future exacerbation risk have produced different results (4, 5). The GOLD 2020 states that there is insufficient evidence to recommend blood eosinophils in stable COPD as a biomarker to predict exacerbation risk (1).

In patients with AECOPD, the clinical value of blood eosinophils in guiding therapy and judging prognosis is less clear. Some studies reported that higher eosinophil levels were associated with shorter hospital stays following treatment with systemic corticosteroids (6, 7), but other studies suggested a longer hospitalization (8). Scarce data exist regarding the association between blood eosinophils and 30-day readmissions or mortality. In the secondary analysis of a randomized controlled trial, there was no difference in treatment failure rates within 30 days between the eosinophilic and non-eosinophilic exacerbations treated with prednisolone (9). Recently, Hegewald et al. reported that greater eosinophil counts were associated with increased risk of 30-day COPD-related readmissions (10). Whether blood eosinophil counts at admission can predict the risk of readmissions and mortality remains controversial. In addition, most of the patients recruited had a history of smoking (6, 7, 9), which might affect the inflammatory response of eosinophils (11). Hence, there is a need for more evidence in clinically representative patient populations.

This real-world study in China aimed to evaluate the association between baseline blood eosinophil counts and the length of stay, systemic corticosteroid usage and healthcare costs, response to systemic corticosteroid therapy during hospitalization in patients with AECOPD. We also investigated whether eosinophil levels are related to the change of COPD Assessment Test (CAT) scores and readmissions or death after 30 days. In addition, clinical outcomes for different eosinophil groups were also assessed according to smoking history.

## Methods

### Study Design and Study Population

We analyzed data from the acute exacerbation of chronic obstructive pulmonary disease inpatient registry (ACURE) study, which was launched to investigate the clinical features, treatments, and prognoses of AECOPD in the Chinese population. Details of the ACURE study have been previously described (12). In brief, the ACURE study is an ongoing nationwide multicenter, observational patient registry in patients admitted for AECOPD, followed up with a 3-year observing period in a real-world setting. Data collected between January 2018 and December 2019 in 163 centers were first reviewed. Only patients ≥18 years old with the discharge diagnosis of an exacerbation of COPD were included. Exacerbations were defined as an acute worsening of respiratory symptoms that result in additional therapy (1). This protocol was approved by the ethics committee of China-Japan Friendship Hospital (No. 2015-88). Informed consent was obtained from all participants in the study. This study was conducted in accordance with the Declaration of Helsinki.

**Figure 1:**
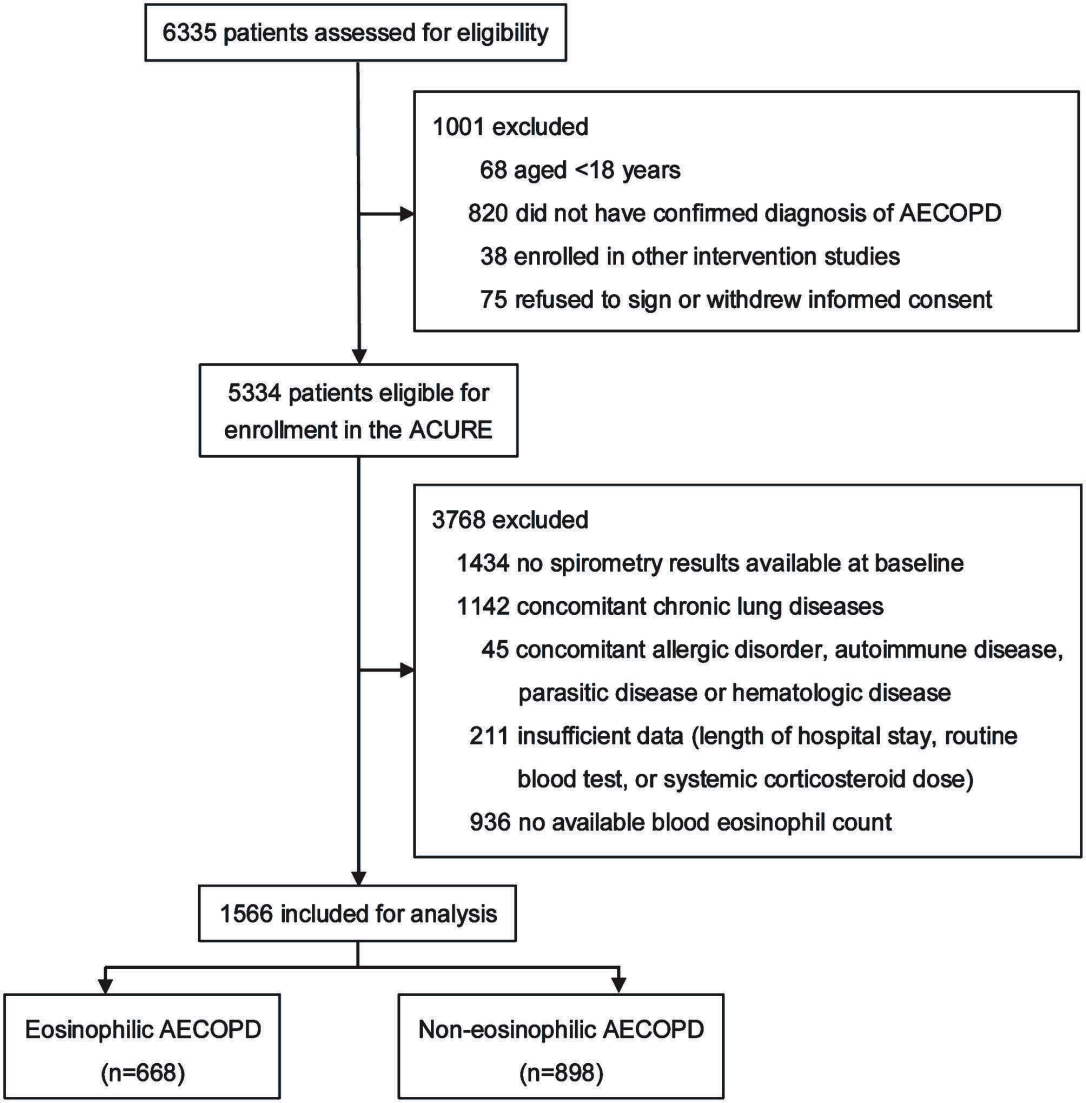
Flowchart of patient enrollment process. AECOPD, acute exacerbations of chronic obstructive pulmonary disease; ACURE, acute exacerbation of chronic obstructive pulmonary disease inpatient registry study.

In our study, the presence of a post-bronchodilator forced expiratory volume in 1 s (FEV1)/forced vital capacity (FVC) ratio <0.70 was required. Patients with a history of asthma, bronchiectasis, pulmonary embolism, pulmonary hypertension, interstitial pulmonary disease, lung cancer or other chronic lung diseases, and those with any comorbidity that could influence blood eosinophil count (allergic disorder, autoimmune disease, parasitic disease or hematologic disease) were excluded. All the included patients had documented blood eosinophil counts on admission. The subject enrollment process is shown in Figure 1. The eligible study population was divided into eosinophilic AECOPD group and non-eosinophilic AECOPD group. The eosinophilic AECOPD was defined as one where the blood eosinophil count at admission was ≥2% of the total white blood cell count, since this threshold had previously shown a sensitivity of 90% and specificity of 60% for predicting sputum eosinophilia in AECOPD (13). In addition, all the included patients were grouped according to their smoking history for further analyzing the effect of smoking on the effectiveness of eosinophils as a biomarker.

### Measurements and Outcomes

Patient baseline characteristics, including age, sex, body-mass index (BMI), smoking status, pulmonary function, symptoms, modified Medical Research Council (mMRC) dyspnea grade, CAT score, the number of hospital or emergency admissions in the previous year, pre-admission medications, complications (respiratory failure and chronic cor pulmonale), comorbidities (pneumonia, hypertension, cardiac disease, diabetes, and cerebrovascular accident), blood eosinophil count, length of hospital stay, hospital corticosteroid treatment, and cost during hospitalization, were recorded. Among these variables, pulmonary function, symptoms, mMRC dyspnea grade, CAT score, respiratory failure, and pneumonia were defined upon admission. The number of hospital or emergency admissions in the previous year, pre-admission medications, chronic cor pulmonale, hypertension, cardiac disease, diabetes, and cerebrovascular accident were defined according to the historical clinical records. Specifically, respiratory failure was defined by an arterial oxygen tension (PaO2) of <8.0 kPa (60 mmHg), an arterial carbon dioxide tension (PaCO2) of >6.0 kPa (45 mmHg) or both (14). The diagnosis of pneumonia was based on the presence of certain clinical features (e.g., cough, fever, sputum production, and pleuritic chest pain) and changes on the chest radiography, which was also supported by pathogenic examination (15). In our study, cardiac disease included coronary heart disease, arrhythmia, chronic heart failure, and acute heart failure. Systemic corticosteroid use was expressed as equivalent dose of prednisolone. The primary outcome was the length of hospital stay. Secondary outcomes were cumulative systemic corticosteroid dose and cost during hospitalization, the change of CAT scores from baseline to discharge or day 30 after discharge, the early readmission with AECOPD or death, taken as those occurring in the 15 days following discharge, and readmission with AECOPD or death within 30 days. The costs were shown in US dollars using the average exchange rate in 2019 (one US dollar was equivalent to 6.90 Chinese yuan).

### Subgroup Analyses

To further examine the response of eosinophils to systemic corticosteroid therapy during hospitalization, subgroup analyses of the primary outcome were performed according to if receiving hospital systemic corticosteroid therapy. In addition, all these analyses were conducted among patients who did not receive systemic corticosteroids prior to admission.

### Statistical Analyses

Continuous data were expressed as median (interquartile range, IQR) and mean (standard error of mean, SEM), and categorical data were expressed as frequencies (percentage). The study variables were compared between eosinophilic and non-eosinophilic AECOPD groups using the chi-squared test for unordered categorical variables and the Mann-Whitney *U*-test for ordinal categorical variables. For continuous variables, the *t*-test and Mann-Whitney *U*-test were used to compare the mean and median respectively.

Given differences in the baseline characteristics between eosinophilic and non-eosinophilic AECOPD groups, propensity score (PS) matching was used to reduce potential bias. We calculated PS using a logistic regression model and used 1:1 matching with a caliper width equal to 0.02 of the standard deviation of the logit of the PS. A PS matching was performed in overall patients included in this study using the following covariates: age, sex, BMI, smoking status, mMRC dyspnea grade, respiratory failure, and pneumonia (forcing age and sex in the model but all other variables were considered only if they reached statistical significance). Further, PS matching was also carried out in patients with smoking history and patients without smoking history, respectively to balance the distributions between eosinophilic and non-eosinophilic AECOPD groups for age, sex, BMI, mMRC dyspnea grade, respiratory failure, and pneumonia.

We used Cox's proportional hazard model to evaluate the hazard ratio between eosinophil level and readmission with AECOPD or death within 15 or 30 days. The potential prognostic factors including, age, sex, BMI, post-bronchodilator FEV_1_/FVC, mMRC dyspnea grade, CAT score, the number of hospital or emergency admissions in the previous year, respiratory failure, pneumonia, and hospital systemic corticosteroid therapy were adjusted in the model. All analyses were performed using the software IBM-SPSS statistics 25. A two-sided *P*-value < 0.05 was considered statistically significant.

## Results

### Baseline Characteristics

Of the 1,566 subjects included in the study, 668 (42.7%) had an eosinophilic AECOPD, whereas 898 subjects (57.3%) had a non-eosinophilic AECOPD. The mean age of the patients was 69 years and 80.3% were male. Most patients had a history of smoking (72.9%) and 27.1% were never smokers. Hypertension was the most common comorbidity.

Baseline characteristics of the eosinophilic and non-eosinophilic groups are shown in Table 1. The mMRC dyspnea grade was significantly lower in eosinophilic compared to non-eosinophilic AECOPD (*P* = 0.005). Prescription of ICS or oral corticosteroids (OCS) before admission was similar between the two groups. Patients in the non-eosinophilic group had higher frequencies of respiratory failure and pneumonia compared with those in the eosinophilic group.

**Table 1:** Clinical characteristics of the study patients before and after propensity score matching.

**Variables**	**Before propensity score matching**			**After propensity score matching**		
	**Eosinophilic AECOPD (*n* = 668)**	**Non-eosinophilic AECOPD (*n* = 898)**	***P*-value**	**Eosinophilic AECOPD (*n* = 650)**	**Non-eosinophilic AECOPD (*n* = 650)**	***P*-value**
Age (years)	69 (63–76)	69 (64–76)	0.280	69 (63–76)	69 (63–76)	0.810
Male	555 (83.1%)	702 (78.2%)	0.016	537 (82.6%)	544 (83.7%)	0.604
Body-mass index (kg/m^2^)	22.5 (19.8–24.6)	21.5 (19.4–24.2)	0.006	22.5 (19.7–24.5)	21.9 (19.6–24.6)	0.496
Smoking status			0.016			0.727
Ex-smoker	319 (47.8%)	370 (41.2%)		309 (47.5%)	286 (44.0%)	
Current smoker	171 (25.6%)	282 (31.4%)		164 (25.2%)	206 (31.7%)	
Non-smoker	178 (26.6%)	246 (27.4%)		177 (27.2%)	158 (24.3%)	
Post-bronchodilator FEV_1_/FVC	0.5 (0.4–0.6)	0.5 (0.4–0.6)	0.893	0.5 (0.4–0.6)	0.5 (0.4–0.6)	0.924
**Symptoms**						
Increased cough	400 (59.9%)	569 (63.4%)	0.160	396 (60.9%)	400 (61.5%)	0.820
Increased sputum volume	262 (39.2%)	397 (44.2%)	0.054	258 (39.7%)	284 (43.7%)	0.144
Increased sputum purulence	273 (40.9%)	406 (45.2%)	0.086	267 (41.1%)	290 (44.6%)	0.197
Wheezing	561 (84.0%)	755 (84.1%)	0.960	551 (84.8%)	545 (83.8%)	0.647
mMRC dyspnea grade			0.005			0.588
0–1	119 (17.8%)	114 (12.7%)		102 (15.7%)	95 (14.6%)	
≥2	549 (82.2%)	784 (87.3%)		548 (84.3%)	555 (85.4%)	
CAT score			0.644			0.704
<10	63 (9.4%)	91 (10.1%)		59 (9.1%)	63 (9.7%)	
≥10	605 (90.6%)	807 (89.9%)		591 (90.9%)	587 (90.3%)	
Hospital admissions previous year			0.436			0.658
0	342 (51.2%)	483 (53.8%)		333 (51.2%)	344 (52.9%)	
1	169 (25.3%)	206 (22.9%)		164 (25.2%)	153 (23.5%)	
≥2	157 (23.5%)	209 (23.3%)		153 (23.5%)	153 (23.5%)	
Emergency visits previous year			0.811			0.645
0	434 (65.0%)	578 (64.4%)		424 (65.2%)	413 (63.5%)	
1	103 (15.4%)	141 (15.7%)		98 (15.1%)	111 (17.1%)	
≥2	131 (19.6%)	179 (19.9%)		128 (19.7%)	126 (19.4%)	
**Pre-admission medication**						
LABA	224 (33.5%)	275 (30.6%)	0.222	220 (33.8%)	220 (33.8%)	1.000
LAMA	240 (35.9%)	293 (32.6%)	0.173	236 (36.3%)	231 (35.5%)	0.773
ICS	226 (33.8%)	279 (31.1%)	0.247	223 (34.3%)	224 (34.5%)	0.953
OCS	17 (2.5%)	28 (3.1%)	0.502	17 (2.6%)	19 (2.9%)	0.735
**Complications**						
Respiratory failure	123 (18.4%)	211 (23.5%)	0.015	123 (18.9%)	131 (20.2%)	0.576
Chronic cor pulmonale	100 (15.0%)	144 (16.0%)	0.565	98 (15.1%)	90 (13.8%)	0.528
**Comorbidities**						
Pneumonia	157 (23.5%)	288 (32.1%)	<0.001	157 (24.2%)	166 (25.5%)	0.563
Hypertension	239 (35.8%)	300 (33.4%)	0.329	234 (36.0%)	217 (33.4%)	0.322
Cardiac disease	140 (21.0%)	195 (21.7%)	0.718	140 (21.5%)	116 (17.8%)	0.094
Diabetes	56 (8.4%)	90 (10.0%)	0.270	56 (8.6%)	67 (10.3%)	0.297
Cerebrovascular accident	46 (6.9%)	62 (6.9%)	0.989	46 (7.1%)	44 (6.8%)	0.827
Length of hospital stay (days)	9 (7–11)	9 (7–12)	0.094	9 (7–11)	9 (7–12)	0.166
Antibiotics during hospitalization	553 (82.8%)	770 (85.7%)	0.109	545 (83.8%)	552 (84.9%)	0.593
Systemic corticosteroids during hospitalization	457 (68.4%)	677 (75.4%)	0.002	447 (68.8%)	489 (75.2%)	0.009
Cumulative systemic corticosteroid dose (mg)	90 (0–200)	120 (0–260)	<0.001	90 (0–200)	120 (0–270)	<0.001
Total cost during hospitalization (US$)	1,333 (1,013–1,842)	1,451 (1,074–2,034)	0.002	1,333 (1,017–1,846)	1,472 (1,086–2,041)	0.002

*Data are presented as n (%) or median (IQR)*.

*AECOPD, acute exacerbations of chronic obstructive pulmonary disease; FEV_1_, forced expiratory volume in 1 s; FVC, forced vital capacity; mMRC, modified Medical Research Council; CAT, COPD Assessment Test; LABA, long-acting beta-adrenoceptor agonist; LAMA, long-acting muscarinic receptor agonist; ICS, inhaled corticosteroids; OCS, oral corticosteroid*.

During hospitalization, the cumulative systemic corticosteroid dose and total healthcare cost were lower in the eosinophilic group than in the non-eosinophilic group (*P* <0.001 and *P* = 0.002, respectively). There was no between-group difference for the primary outcome of length of hospital stay (*P* = 0.094; Table 1).

### Propensity Score Matching Analyses

Of all included patients, 650 of 668 eosinophilic cases could be matched (1:1) to a non-eosinophilic control. After matching, the two groups were well-balanced regarding all baseline covariates. Notably, results from the PS matching analyses were similar to those from the unmatched analyses. The median hospitalization days of the two groups were both 9 days with no statistical difference (*P* = 0.166).

**Figure 2:**
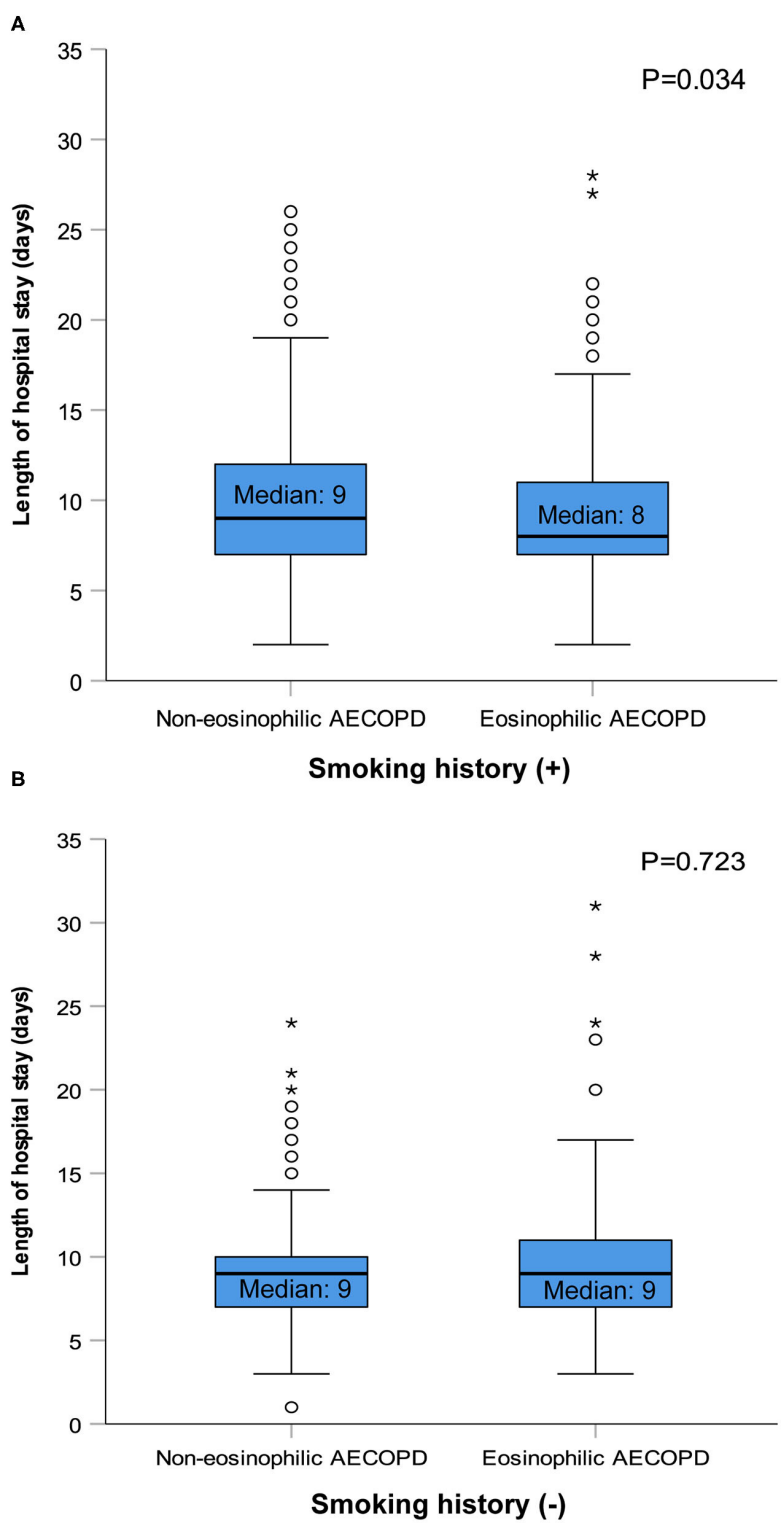
Length of hospital stay in patients with or without smoking history after matching. AECOPD, acute exacerbations of chronic obstructive pulmonary disease. The circles and asterisks are outliers.

**Figure 3:**
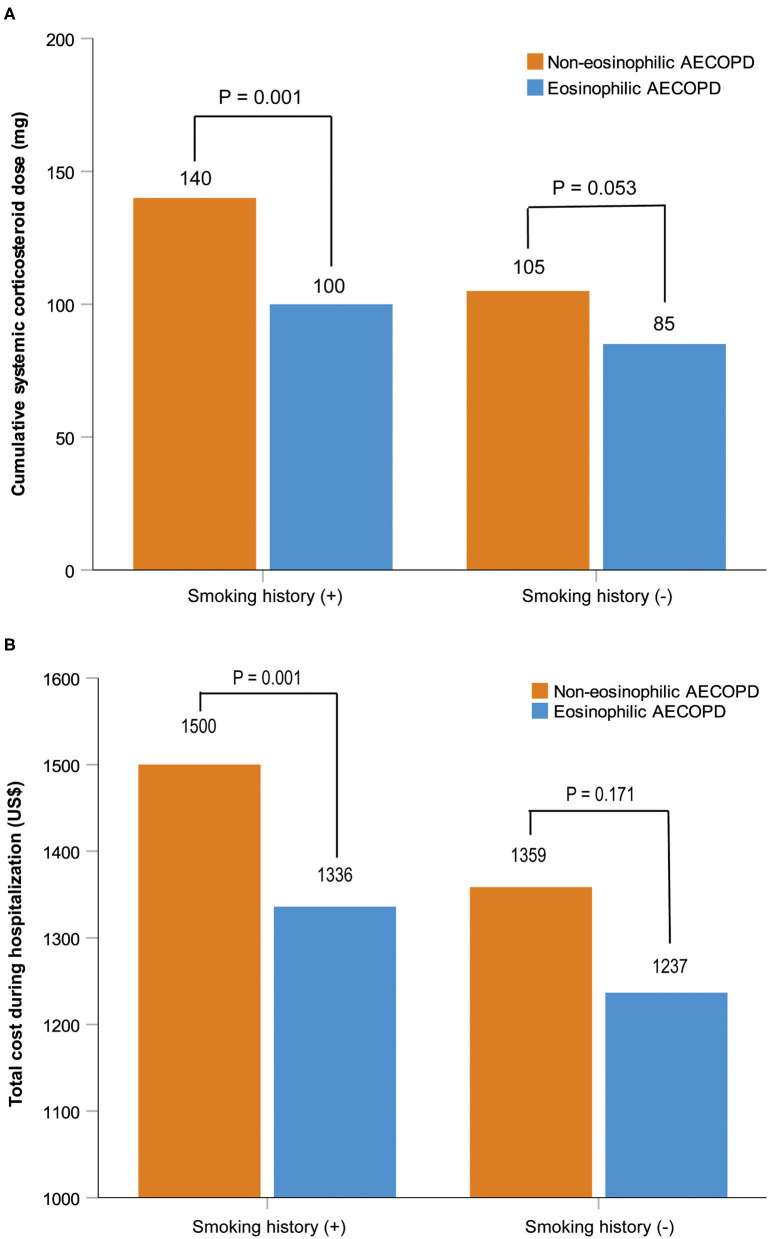
Cumulative systemic corticosteroid dose and total cost during hospitalization in patients with or without smoking history after matching. Data are presented as median. AECOPD, acute exacerbations of chronic obstructive pulmonary disease.

**Figure 4:**
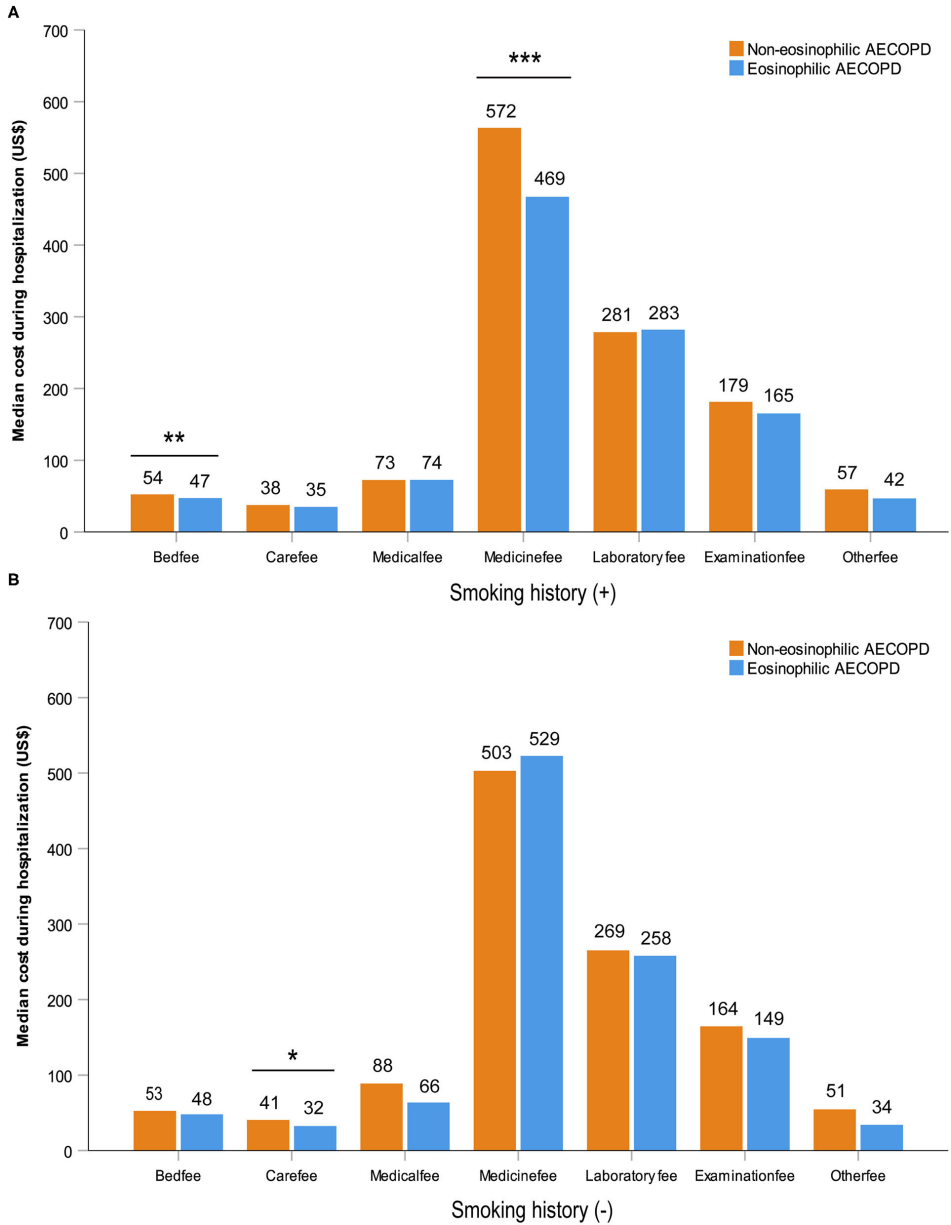
Various cost during hospitalization in patients with or without smoking history after matching. Statistically significant differences between groups are indicated as *P ≤ 0.05, **P ≤ 0.01, and ***P ≤ 0.001. Error bars show 95% confidence interval. AECOPD, acute exacerbations of chronic obstructive pulmonary disease.

After performing PS matching for the 1,142 patients with a history of smoking in the entire 1,566 subjects, 468 matched pairs of patients were selected. In the 424 patients without smoking history, 177 matched pairs were identified. The distribution of baseline characteristics was balanced after matching between the eosinophilic and non-eosinophilic groups in patients with or without smoking history (Supplementary Table 1). For patients with smoking history, the median time in hospital of the eosinophilic group was significantly shorter compared with the non-eosinophilic group [median (IQR) 8 days (7–11) vs. 9 days (7–12), *P* = 0.034; Figure 2A], whereas no statistical difference was found between the two groups in patients who were never smokers (Figure 2B). The total dosage of systemic corticosteroids and total cost during hospitalization were lower in eosinophilic exacerbations than in non-eosinophilic exacerbations among patients with smoking history (*P* = 0.001 for both; Figure 3), but the differences were not statistically significant in never smokers (*P* = 0.053 and 0.171, respectively; Figure 3). Accounting for the highest proportion of total cost, medicine fee in patients with smoking history was also numerically higher for the non-eosinophilic group, as compared with the eosinophilic group (Figure 4A). However, in never smokers, the eosinophilic group exhibited higher medicine fee, although the result was not statistically significant (Figure 4B). Antibiotics use during hospitalization showed no difference between the groups regardless of smoking history.

### Subgroup Analyses

In subgroup analyses of the matched cohorts, systemic corticosteroids were prescribed in 72.0, 74.6, and 66.7% of the overall patients, the patients with smoking history and the patients with no smoking history, respectively (Supplementary Table 2). Length of hospital stay showed no significant difference between the eosinophilic and non-eosinophilic groups no matter whether using systemic corticosteroids or not in overall patients (*P* = 0.392 and 0.245, respectively). The hospitalization time following treatment with systemic corticosteroids was shorter in eosinophilic AECOPD compared to non-eosinophilic AECOPD in patients with smoking history [median (IQR) 8 days (7–12) vs. 9 days (7–12), *P* = 0.046] but similar between the two groups in patients with no history of smoking (*P* = 0.376). There was no statistical difference in length of hospital stay between the two groups in patients not receiving corticosteroids therapy, regardless of smoking history (*P* = 0.520 and 0.494, respectively). Limiting the analysis to patients free of OCS use prior to admission did not change these results (Supplementary Table 3).

### Longitudinal Analyses

**Figure 5:**
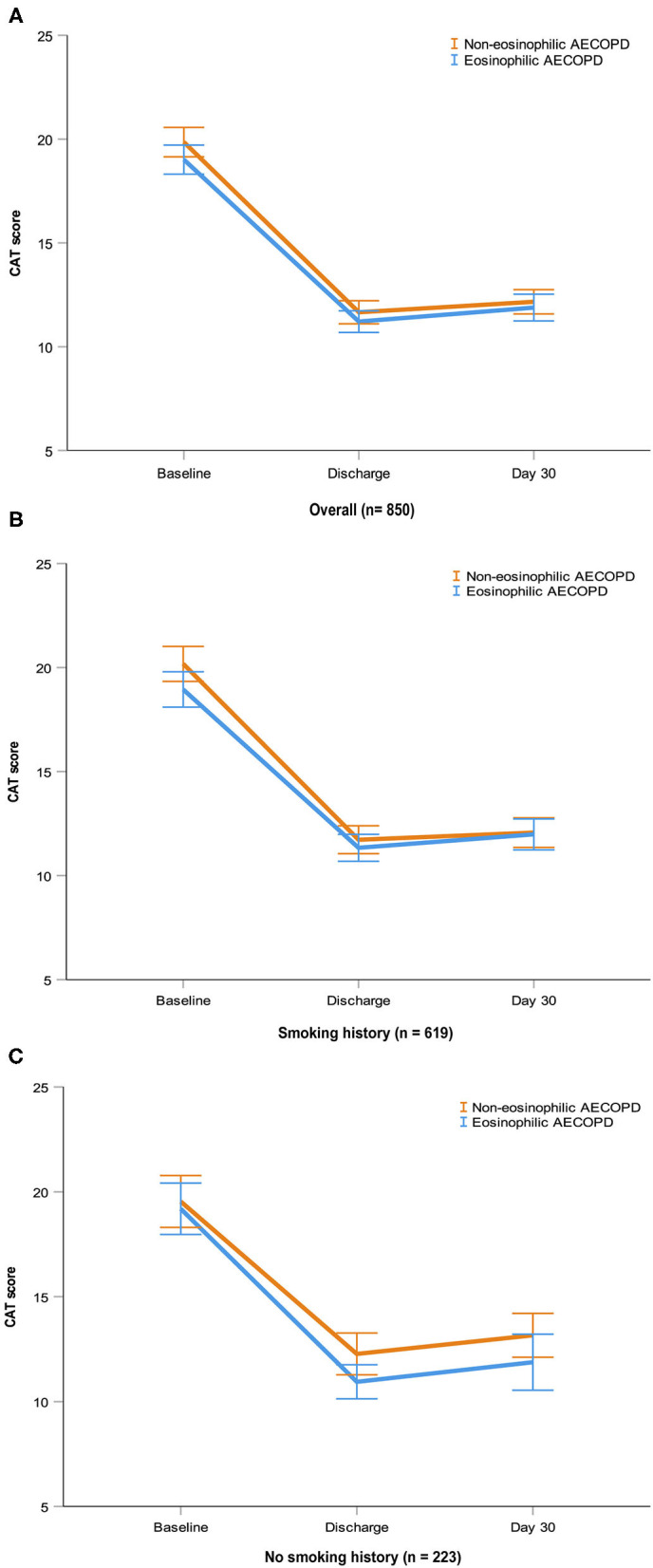
CAT scores at baseline, discharge and day 30 in the three matched cohorts. Data are presented as mean (SEM). AECOPD, acute exacerbations of chronic obstructive pulmonary disease; CAT, COPD Assessment Test; SEM, standard error of mean.

A total of 1,003 patients (64%) of 1,566 had follow-up data within 30 days after discharge. Clinical characteristics were largely similar among subjects with and without follow-up data (Supplementary Table 4). Readmission with AECOPD or death occurred in 1.6 and 3.5% of the 1,003 patients at 15 and 30 days, respectively. Cox regression analyses showed, in overall patients or patients with smoking history after matching, there was no significant difference in 15-day readmission or death between the eosinophilic and non-eosinophilic groups, nor was there a difference between the groups at 30 days (Table 2). Given the small number of index events in the non-smoking cohort, no further analysis was undertaken. At all timepoints evaluated, the mean change from the baseline in CAT scores did not differ between the two groups in the three matched cohorts (Figure 5).

**Table 2:** Readmission with AECOPD or death within 15 and 30 days in the matched cohorts.

	**Readmission with AECOPD or death within 15 days**		**Readmission with AECOPD or death within 30 days**	
	**HR (95% CI)^a^**	***P*-value**	**HR (95% CI)^a^**	***P*-value**
**Overall (*****n*** **=** **850)**				
Non-eosinophilic AECOPD	1 (Ref)		1 (Ref)	
Eosinophilic AECOPD	1.002 (0.300–3.346)	0.998	1.264 (0.572–2.793)	0.562
**Smoking history (*****n*** **=** **619)**				
Non-eosinophilic AECOPD	1 (Ref)		1 (Ref)	
Eosinophilic AECOPD	1.034 (0.267–4.005)	0.962	1.064 (0.425–2.665)	0.895

a *Cox proportional hazards model*.

*AECOPD, acute exacerbations of chronic obstructive pulmonary disease*.

## Discussion

To the best of our knowledge, this has been the first multicenter observational study using propensity matching analysis of real-world data to evaluate the role of eosinophils in patients with AECOPD requiring hospitalization. We found that the increased eosinophil count was associated with a better short-term prognosis only in patients with smoking history. For patients who were never smokers, the predict value of eosinophils was poor. In addition, we for the first time proposed that blood eosinophil counts might be a useful biomarker for economic burden of AECOPD during hospitalization. The nature of the exacerbation was not relevant to the change of CAT scores and readmissions for AECOPD or death after 30 days.

The prevalence of elevated blood eosinophils, defined as ≥2% of the total leukocyte count, was about 40% in our study, similar to recent studies in China (6, 16). According to this threshold, inpatients with eosinophilic AECOPD may have different clinical characteristics. In our study, lower eosinophil level was associated with a higher risk of respiratory failure and higher mMRC dyspnea grade. Another multicenter prospective study of 493 AECOPD patients also reported that patients with lower eosinophil counts experienced higher rates of both non-invasive mechanical ventilation and respiratory failure (17). Moreover, we found a strong relationship between low levels of eosinophils and the development of pneumonia. In a prospective observational cohort study, Kim et al. showed that eosinophils ≥2% at exacerbation was associated with a lower risk of bacterial infections (18). C-reactive protein as a proxy for infection correlated negatively with blood eosinophil counts (19).

In our real-world study, the difference of hospitalization time was not significant between the eosinophilic and non-eosinophilic exacerbations. The PS matching analyses did not change this result. Only in patients with a history of smoking, eosinophils ≥2% distinguished those with shorter median hospital stays. As we know, airway inflammation and remodeling are closely related to cigarette smoking, which is the most commonly encountered risk factor for COPD (1). However, the molecular mechanisms are not completely clear. The mechanism of inflammation in patients developing COPD without smoking is also less known. A growing number of researchers regarded eosinophils as a biomarker and proposed that eosinophilic inflammation was a common and stable phenotype in COPD (6, 16–19). It is necessary to consider the influence of smoking on eosinophilic inflammation. Chis et al. reported that the level of blood eosinophils was affected by smoking status in COPD patients (20). In another study that assessed the accuracy of inflammatory biomarkers in differentiating asthma-COPD overlap (ACO) from COPD, blood eosinophil counts had a higher sensitivity in distinguishing ACO from COPD among patients with a smoking history compared to those without smoking (21).

Many researches showed that patients presenting to hospital with an eosinophilic AECOPD had a shorter length of stay. However, a large number of these studies included patients who were former or current smokers with a minimum smoking history of 10 pack years (7, 19, 22–24). In several other studies, non-smokers were included but occupied a little proportion (<5%) (6, 25). Similar to our study, Gonzalez-Barcala et al. carried out a retrospective study including 358 patients in Spain, 26.5% of whom were never smokers. They found no significant differences in the length of stay between patients with an elevated or low blood eosinophil counts using various thresholds (26). In a study performed in three teaching hospitals in China, a longer hospital stay was observed in the non-eosinophilic group although 36.5% of the included patients had no history of smoking (17). In fact, patients with lower eosinophil counts in this study experienced a higher rate of infection indicated by the higher leukocyte counts and neutrophil percentages at baseline, which also leading to longer hospital stays.

In our study, higher eosinophil levels correlated with a better response to hospital systemic corticosteroid therapy only in patients with smoking history. The association between hospital stay and blood eosinophil levels became non-statistically significant in the whole sample or in patients without smoking following treatment with corticosteroids. Previous related studies also reported that patients with non-eosinophilic exacerbations were less responsive to systemic corticosteroids, noting that almost all of the included patients had a history of smoking (6, 7, 9, 23). Few researchers have considered smoking status when studying eosinophils and the response to corticosteroids. In *The Lancet Respiratory Medicine*, Bafadhel et al. reported that smoking status was an independent predictor of response to budesonide in COPD patients in a *post-hoc* analysis of three trials and a significant interaction was observed between eosinophil count, treatment, and smoking status (27). They found in former smokers, exacerbation rate was independent of eosinophil count, but in current smokers, increased eosinophil counts were associated with increased risk of exacerbation, with a treatment effect of budesonide–formoterol as compared with formoterol alone associated with elevated eosinophil level. A *post-hoc* supervised cluster analysis showed a favorable response to fluticasone furoate/vilanterol compared with vilanterol alone in COPD patients with higher eosinophil counts or lower eosinophil counts with shorter smoking history (28). The complicated mechanisms underlying the interaction between smoking status and corticosteroid benefit remain unclear. However, in our study, never smokers had better lung function and a lower proportion of pre-admission medication usage than patients with a history of smoking. They might be more sensitive to medications like bronchodilators and antibiotics and have better compliance in the management of AECOPD, which made the differences in clinical outcomes between the eosinophilic and non-eosinophilic groups insignificant. Cigarette smoke may directly affect the response of eosinophils to corticosteroids, but the molecular mechanism and related pathway need to be further studied.

In addition, the higher dose of systemic corticosteroids as well as the number of patients treated with systemic corticosteroids in the non-eosinophilic group in our study might be due to their poorer response to corticosteroid therapy. More non-eosinophilic patients were prescribed systemic corticosteroids rather than inhaled corticosteroids and the dose of systemic corticosteroids needed in the non-eosinophilic AECOPD was also higher.

Several studies demonstrated a substantial annual burden associated with eosinophilic COPD (29, 30). In a cross-sectional study of 2,832 patients in the US, subjects with elevated eosinophil counts had numerically higher all-cause and COPD-related health care resource utilization (HCRU) and cost each year (31). However, there has been no study showing the relationship between eosinophil counts and cost during an exacerbation in hospitalized patients. Our study found that blood eosinophil counts could serve as a biomarker for the total cost during hospitalization in patients with AECOPD. The higher medicine fee in the non-eosinophilic group among patients with smoking history might be related to their higher dosage of corticosteroid use and longer hospital stay.

In our study, no relationship was found between eosinophil counts and clinical outcomes after 15 or 30 days. Gonzalez-Barcala et al. also reported that there was no relationship observed between eosinophil counts and readmissions within 15 days (26). Using data from a randomized clinical trial, Prins et al. claimed that treatment failure within 10 days was reduced in the eosinophilic group, but late treatment failure (days 11–30) did not differ significantly (22). In a randomized biomarker-directed corticosteroid vs. standard therapy study, the mean change from the baseline in CAT scores and readmissions with AECOPD or death at 30 days did not differ between the eosinophilic and non-eosinophilic groups (32). However, in another similar study, there was a greater improvement of the chronic respiratory questionnaire over 14 days in the eosinophilic exacerbations treated with prednisolone (9). Of note, the majority of the exacerbations in this study did not require hospitalization and the dosage of prednisolone in this study (30 mg once daily for 14 days) was higher than that in the real world. Recently, Hegewald et al. reported that greater eosinophil counts were associated with increased risk of COPD-related readmission at 30 days, but 26% of the included patients combined asthma and only 17.6% had spirometry results, which were likely to influence the outcome (10).

The strength of this study was that it represented a real-world study of a large AECOPD cohort, which allowed the impact of smoking status to be discerned. Moreover, the propensity matching technique minimized bias by comparing groups with similar baseline characteristics. All patients had a diagnosis of COPD confirmed by spirometry. The present study also had several limitations. First, we used the percentage of eosinophils for analysis, which could be affected by the increased neutrophil count during exacerbations. Moreover, only the blood eosinophil count upon admission was recorded, but this value might be variable over time. Second, not all patients included in this study had follow-up data after 30 days. However, the baseline characteristics between those with or without follow-up data showed no significant differences, making selective bias less likely. Third, to date, we did not have long-term follow-up data because the ACURE study is an ongoing study with only the 30-day data available. But waiting for long-term follow-up would have delayed the reporting of these important results for several years. Fourth, only about 40% of the patients eligible for the ACURE study were recruited and we cannot be sure that our study can be implemented with the same result in patients not recruited. Finally, although PS matching was used to adjust for known baseline variables, the confounders from unmeasured variables could also influence the results.

## Conclusion

This study found that for patients hospitalized for AECOPD with a history of smoking, eosinophil count <2% was associated with a longer length of hospital stay, a higher dosage of corticosteroid use, a higher economic burden of hospitalization, and a poor response to corticosteroid therapy. However, no significant difference was found in patients without smoking. Eosinophil levels cannot be confidently used as a predictor alone for short-term prognosis and have no relationship with 30-day clinical outcomes. Further studies are needed to assess the effect of smoking or other factors on the efficacy of eosinophils as a biomarker.

## Data Availability Statement

The raw data supporting the conclusions of this article will be made available by the authors, without undue reservation.

## Ethics Statement

The studies involving human participants were reviewed and approved by the ethics committee of China-Japan Friendship Hospital. The patients/participants provided their written informed consent to participate in this study.

## Author Contributions

Concept and design of the study by YCu, YCh, TY, CL, and KH. Data collection and management by YCu, ZZh, ZZe, CL, XM, KH, YZ, XR, TY, and YCh. Statistical analysis by YCu, ZZh, CL, XM, and YZ. Drafting of the manuscript by YCu. All authors contributed to the article and approved the submitted version.

## Conflict of Interest

The authors declare that the research was conducted in the absence of any commercial or financial relationships that could be construed as a potential conflict of interest.
