# Supplementary material for: Blood Eosinophils and Clinical Outcomes in Patients With Acute Exacerbation of Chronic Obstructive Pulmonary Disease: A Propensity Score Matching Analysis of Real-World Data in China
Source: Front Med (Lausanne). 2021 Jun 9;8:653777. doi: 10.3389/fmed.2021.653777 (PMC8219875; doi:10.3389/fmed.2021.653777)
Supplement: Supplementary file 1 [file Data_Sheet_1.DOCX]

**Table E1 Baseline characteristics of the patients with or without smoking history after matching**

| **Variables** | **Smoking history** | | | **No smoking history** | | |
| --- | --- | --- | --- | --- | --- | --- |
|  | **Eosinophilic AECOPD (n=468)** | **Non-eosinophilic AECOPD (n=468)** | ***P*-value** | **Eosinophilic AECOPD (n=177)** | **Non-eosinophilic AECOPD (n=177)** | ***P*-value** |
| Age (years) | 68 (62-75) | 69 (63-75) | 0.781 | 71 (65-77) | 70 (65-78) | 0.684 |
| Male | 447 (95.5%) | 448 (95.7%) | 0.873 | 85 (48.0%) | 81 (45.8%) | 0.670 |
| Body-mass index (kg/m^2^) | 22.3 (19.5-24.3) | 21.6 (19.5-24.3) | 0.196 | 22.5 (20.0-25.0) | 22.7 (20.2-25.6) | 0.274 |
| Post-bronchodilator FEV_1_/FVC | 0.5 (0.4-0.6) | 0.5 (0.4-0.6) | 0.535 | 0.6 (0.5-0.6) | 0.6 (0.5-0.6) | 0.590 |
| Symptoms |  |  |  |  |  |  |
| Increased cough | 272 (58.1%) | 290 (62.0%) | 0.230 | 122 (68.9%) | 116 (65.5%) | 0.497 |
| Increased sputum volume | 179 (38.2%) | 206 (44.0%) | 0.073 | 78 (44.1%) | 80 (45.2%) | 0.831 |
| Increased sputum purulence | 184 (39.3%) | 207 (44.2%) | 0.127 | 81 (45.8%) | 78 (44.1%) | 0.749 |
| Wheezing | 401 (85.7%) | 393 (84.0%) | 0.466 | 144 (81.4%) | 150 (84.7%) | 0.395 |
| mMRC dyspnea grade |  |  | 0.853 |  |  | 0.768 |
| 0-1 | 69 (14.7%) | 67 (14.3%) |  | 28 (15.8%) | 26 (14.7%) |  |
| ≥2 | 399 (85.3%) | 401 (85.7%) |  | 149 (84.2%) | 151 (85.3%) |  |
| CAT score |  |  | 0.733 |  |  | 0.711 |
| <10 | 44 (9.4%) | 41 (8.8%) |  | 15 (8.5%) | 17 (9.6%) |  |
| ≥10 | 424 (90.6%) | 427 (91.2%) |  | 162 (91.5%) | 160 (90.4%) |  |
| Hospital admissions previous year |  |  | 0.577 |  |  | 0.816 |
| 0 | 232 (49.6%) | 241 (51.5%) |  | 96 (54.2%) | 96 (54.2%) |  |
| 1 | 127 (27.1%) | 113 (24.1%) |  | 39 (22.0%) | 44 (24.9%) |  |
| ≥2 | 109 (23.3%) | 114 (24.4%) |  | 42 (23.7%) | 37 (20.9%) |  |
| Emergency visits previous year |  |  | 0.850 |  |  | 0.575 |
| 0 | 298 (63.7%) | 296 (63.2%) |  | 120 (67.8%) | 115 (65.0%) |  |
| 1 | 74 (15.8%) | 80 (17.1%) |  | 26 (14.7%) | 28 (15.8%) |  |
| ≥2 | 96 (20.5%) | 92 (19.7%) |  | 31 (17.5%) | 34 (19.2%) |  |
| Pre-admission medication |  |  |  |  |  |  |
| LABA | 167 (35.7%) | 164 (35.0%) | 0.837 | 51 (28.8%) | 52 (29.4%) | 0.907 |
| LAMA | 178 (38.0%) | 171 (36.5%) | 0.636 | 57 (32.2%) | 56 (31.6%) | 0.909 |
| ICS | 169 (36.1%) | 166 (35.5%) | 0.838 | 52 (29.4%) | 52 (29.4%) | 1.000 |
| OCS | 12 (2.6%) | 12 (2.6%) | 1.000 | 5 (2.8%) | 7 (4.0%) | 0.557 |
| Complications |  |  |  |  |  |  |
| Respiratory failure | 87 (18.6%) | 100 (21.4%) | 0.288 | 35 (19.8%) | 36 (20.3%) | 0.894 |
| Chronic cor pulmonale | 76 (16.2%) | 70 (15.0%) | 0.589 | 21 (11.9%) | 21 (11.9%) | 1.000 |
| Comorbidities |  |  |  |  |  |  |
| Pneumonia | 112 (23.9%) | 107 (22.9%) | 0.699 | 45 (25.4%) | 48 (27.1%) | 0.717 |
| Hypertension | 161 (34.4%) | 157 (33.5%) | 0.783 | 68 (38.4%) | 66 (37.3%) | 0.827 |
| Cardiac disease | 95 (20.3%) | 87 (18.6%) | 0.509 | 44 (24.9%) | 41 (23.2%) | 0.709 |
| Diabetes | 42 (9.0%) | 49 (10.5%) | 0.440 | 13 (7.3%) | 19 (10.7%) | 0.266 |
| Cerebrovascular accident | 27 (5.8%) | 36 (7.7%) | 0.240 | 18 (10.2%) | 11 (6.2%) | 0.175 |

Data are presented as n (%) or median (IQR).

AECOPD, acute exacerbations of chronic obstructive pulmonary disease; FEV_1_, forced expiratory volume in 1 s; FVC, forced vital capacity; mMRC, modified Medical Research Council; CAT, COPD Assessment Test; LABA, long-acting beta-adrenoceptor agonist; LAMA, long-acting muscarinic receptor agonist; ICS, inhaled corticosteroids; OCS: oral corticosteroid.

**Table E2 Length of hospital stay in the three matched cohorts according to hospital systemic corticosteroid use**

|  | **Systemic corticosteroid use** | | | **No systemic corticosteroid use** | | |
| --- | --- | --- | --- | --- | --- | --- |
|  | **n** | **Median (IQR)** | ***P*-value** | **n** | **Median (IQR)** | ***P*-value** |
| Overall |  |  | 0.392 |  |  | 0.245 |
| Eosinophilic AECOPD | 447 | 9 (7-11) |  | 203 | 8 (7-11) |  |
| Non-eosinophilic AECOPD | 489 | 9 (7-12) |  | 161 | 9 (7-12) |  |
| Smoking history |  |  | 0.046 |  |  | 0.520 |
| Eosinophilic AECOPD | 336 | 8 (7-12) |  | 132 | 8 (7-11) |  |
| Non-eosinophilic AECOPD | 362 | 9 (7-12) |  | 106 | 9 (7-12) |  |
| No smoking history |  |  | 0.376 |  |  | 0.494 |
| Eosinophilic AECOPD | 112 | 9 (7-11) |  | 65 | 9 (7-12) |  |
| Non-eosinophilic AECOPD | 124 | 9 (7-11) |  | 53 | 9 (8-10) |  |

AECOPD, acute exacerbations of chronic obstructive pulmonary disease.

**Table E3 Length of hospital stay in** **patients free of OCS use prior to admission in the three matched cohorts according to hospital systemic corticosteroid use**

|  | **Total subjects** | | | **Systemic corticosteroid use** | | | **No systemic corticosteroid use** | | |
| --- | --- | --- | --- | --- | --- | --- | --- | --- | --- |
|  | **n** | **Median (IQR)** | ***P*-value** | **n** | **Median (IQR)** | ***P*-value** | **n** | **Median (IQR)** | ***P*-value** |
| Overall |  |  | 0.102 |  |  | 0.254 |  |  | 0.248 |
| Eosinophilic AECOPD | 633 | 8 (7-11) |  | 433 | 9 (7-11) |  | 200 | 8 (7-11) |  |
| Non-eosinophilic AECOPD | 631 | 9 (7-12) |  | 472 | 9 (7-12) |  | 159 | 9 (7-12) |  |
| Smoking history |  |  | 0.011 |  |  | 0.015 |  |  | 0.488 |
| Eosinophilic AECOPD | 456 | 8 (7-11) |  | 326 | 8 (7-11) |  | 130 | 8 (7-10) |  |
| Non-eosinophilic AECOPD | 456 | 9 (7-12) |  | 351 | 9 (7-12) |  | 105 | 9 (7-12) |  |
| No smoking history |  |  | 0.555 |  |  | 0.263 |  |  | 0.561 |
| Eosinophilic AECOPD | 172 | 9 (7-11) |  | 108 | 9 (7-11) |  | 64 | 9 (7-12) |  |
| Non-eosinophilic AECOPD | 170 | 9 (7-10) |  | 118 | 9 (7-10) |  | 52 | 9 (8-10) |  |

OCS, oral corticosteroid; AECOPD, acute exacerbations of chronic obstructive pulmonary disease.

**Table E4 Baseline characteristics of the patients with or without follow-up data**

| **Variables** | **With follow-up data (n=1003)** | **Without follow-up data (n=563)** | ***P*-value** |
| --- | --- | --- | --- |
| Age (years) | 70 (64-76) | 69 (63-76) | 0.221 |
| Male | 808 (80.6%) | 449 (79.8%) | 0.700 |
| Body-mass index (kg/m^2^) | 22.0 (19.6-24.5) | 22.0 (19.2-24.4) | 0.405 |
| Smoking status |  |  | 0.227 |
| Ex-smoker | 440 (43.9%) | 249 (44.2%) |  |
| Current smoker | 303 (30.2%) | 150 (26.6%) |  |
| Non-smoker | 260 (25.9%) | 164 (29.1%) |  |
| Post-bronchodilator FEV_1_/FVC | 0.5 (0.4-0.6) | 0.5 (0.4-0.6) | 0.825 |
| mMRC dyspnea grade | 3 (2-3) | 3 (2-3) | 0.347 |
| CAT score | 19 (14-25) | 20 (15-25) | 0.640 |
| Hospital admissions previous year |  |  | 0.038 |
| 0 | 552 (55.0%) | 273 (48.5%) |  |
| 1 | 232 (23.1%) | 143 (25.4%) |  |
| ≥2 | 219 (21.8%) | 147 (26.1%) |  |
| Emergency visits previous year |  |  | 0.938 |
| 0 | 651 (64.9%) | 361 (64.1%) |  |
| 1 | 145 (14.5%) | 99 (17.6%) |  |
| ≥2 | 207 (20.6%) | 103 (18.3%) |  |
| Pre-admission medication |  |  |  |
| LABA | 326 (32.5%) | 173 (30.7%) | 0.470 |
| LAMA | 343 (34.2%) | 190 (33.7%) | 0.857 |
| ICS | 332 (33.1%) | 173 (30.7%) | 0.335 |
| OCS | 31 (3.1%) | 14 (2.5%) | 0.492 |
| Complications |  |  |  |
| Respiratory failure | 230 (22.9%) | 104 (18.5%) | 0.040 |
| Chronic cor pulmonale | 166 (16.6%) | 78 (13.9%) | 0.158 |
| Comorbidities |  |  |  |
| Pneumonia | 298 (29.7%) | 147 (26.1%) | 0.130 |
| Hypertension | 360 (35.9%) | 179 (31.8%) | 0.101 |
| Cardiac disease | 218 (21.7%) | 117 (20.8%) | 0.659 |
| Diabetes | 94 (9.4%) | 52 (9.2%) | 0.929 |
| Cerebrovascular accident | 75 (7.5%) | 33 (5.9%) | 0.226 |

Data are presented as n (%) or median (IQR).

FEV_1_, forced expiratory volume in 1 s; FVC, forced vital capacity; mMRC, modified Medical Research Council; CAT, COPD Assessment Test; LABA, long-acting beta-adrenoceptor agonist; LAMA, long-acting muscarinic receptor agonist; ICS, inhaled corticosteroids; OCS: oral corticosteroid.
